# Supplementary material for: Performance of volunteer community health workers in implementing home-fortification interventions in Bangladesh: A qualitative investigation
Source: PLoS One. 2020 Apr 1;15(4):e0230709. doi: 10.1371/journal.pone.0230709 (PMC7112190; doi:10.1371/journal.pone.0230709)
Supplement: S1 Table — (DOCX) [file pone.0230709.s002.docx]

**Table S1: An additional table to describe number of interviews conducted under each data collection techniques**

| **Data collection techniques** | **Participants** | **Number of interviews/FGDs** | **Number of participants** |
| --- | --- | --- | --- |
| In-depth interview | Shasthya Shebika | 15 | 15 |
|  | Caregivers | 22 | 22 |
|  | Shasthya Kormi (if any) | 32 | 32 |
|  | SS Husband | 2 | 2 |
| FGD | Shasthya Shebika | 10 | 54 |
|  | Shasthya Kormi | 3 | 24 |
| Key informant interview | Program Organizer | 17 | 17 |
|  | District Manager | 4 | 4 |
|  | Upazila Manager | 9 | 9 |
|  | Supply Chain Officer | 4 | 4 |
|  | Upazila Health and Family Planning Officer | 2 | 2 |
|  | Village Doctor | 1 | 1 |
| Total |  |  | 186 |
