# Supplementary material for: Performance of volunteer community health workers in implementing home-fortification interventions in Bangladesh: A qualitative investigation
Source: PLoS One. 2020 Apr 1;15(4):e0230709. doi: 10.1371/journal.pone.0230709 (PMC7112190; doi:10.1371/journal.pone.0230709)
Supplement: Attachment — Submitted filename: Review round 2.docx [file pone.0230709.s004.docx]

**General Comments**

The authors have made a concerted effort to address the comments raised. Additional reviewers for grammar and spelling should be done.

**Specific sections**

**Abstract**

1. Line 18 should be receive instead of received.
2. Line 22 consider revising the use of “during the period of June 2014 to December 2016 “ to “between the period of June 2014 to December 2016. “

**Introduction**

1. Line 93: Consider using between rather than during.
2. Line 110. Revise the structure of the sentence. Are you stating that the performance is measured by various factors or that it is shaped by various factors?

**Methods**

1. Line 126. The abbreviation for community health workers is wrong.
2. Line 138-143. Authors use of triangulation as described here refers more to synthesis of the data from the quantitative and qualitative evaluations rather than the different qualitative approaches. As such it is not entirely relevant for the description of this particular study. They should consider revising this to reflect that.
3. Lines 203-210. This section should come earlier. Before the paragraph that starts at line 186.
4. Though authors have made some changes to the section, however could the consider breaking it into subsections so that it is somewhat easier to read.
