# Supplementary material for: Performance of volunteer community health workers in implementing home-fortification interventions in Bangladesh: A qualitative investigation
Source: PLoS One. 2020 Apr 1;15(4):e0230709. doi: 10.1371/journal.pone.0230709 (PMC7112190; doi:10.1371/journal.pone.0230709)
Supplement: Attachment — Submitted filename: Response to Reviewers_Revision round 2.docx [file pone.0230709.s005.docx]

**Performance of Volunteer Community Health Workers in Implementing Home-fortification Interventions in Bangladesh: A Qualitative Investigation**

**Revision round two**

**Reviewer comments and our responses**

**Reviewer #1: General Comments**

The authors have made a concerted effort to address the comments raised. Additional reviewers for grammar and spelling should be done.

Specific sections

Abstract
1. Line 18 should be receive instead of received.

Response: Revised as suggested

2. Line 22 consider revising the use of “during the period of June 2014 to December 2016 “ to “between the period of June 2014 to December 2016. “

Response: Revised as suggested

Introduction
1. Line 93: Consider using between rather than during.

Response: Revised as suggested

2. Line 110. Revise the structure of the sentence. Are you stating that the performance is measured by various factors or that it is shaped by various factors?

Response: Revised as suggested

Methods
1. Line 126. The abbreviation for community health workers is wrong.

Response: Thanks, corrected it accordingly

2. Line 138-143. Authors use of triangulation as described here refers more to synthesis of the data from the quantitative and qualitative evaluations rather than the different qualitative approaches. As such it is not entirely relevant for the description of this particular study. They should consider revising this to reflect that.

Response: Considering reviewer concerns we have revised this sentence.

3. Lines 203-210. This section should come earlier. Before the paragraph that starts at line 186.

Response: Agreed and rearranged the section as suggested.

4. Though authors have made some changes to the section, however could the consider breaking it into subsections so that it is somewhat easier to read.

Response: We split the final paragraph of method section into two paragraphs.

**Reviewer #2: Minor edits:**

Line 141 - "validity of estimates", what does this mean? You are presenting qualitative findings, unclear on what estimates you are referring to?

Response: Revised

Line 184 - change, "caregivers who never used MNP"

Response: Changed as suggested

Line 257 - "consisted of two"

Response: Revised

Line 260 - "responded to all queries"

Response: Revised

Line 261 - "asked them to give consent"

Response: Revised.

Line 282 - "An SS completed on average, 4 years of schooling"

Response: Revised.

Line 383 - "sometimes community members did not accept..."

Response: Revised.

Line 631 - "should review existing pay"

Response: Revised.
